# Successful retrieval of dislocated inferior vena cava filter using double vascular sheaths docking technology: case report

**DOI:** 10.1186/s12959-021-00309-3

**Published:** 2021-08-17

**Authors:** Mi Zhou, Lixing Qi, Yongquan Gu

**Affiliations:** Department of Vascular Surgery, Xuanwu Hospital, Capital Medical University , No. 45 Changchun Street, Xicheng District, 100053 Beijing, P. R. China

**Keywords:** IVCF, Dislocation, Deep vein thrombosis

## Abstract

**Background:**

Dislocation of inferior vena cava filter (IVCF) is a rare complication with potential IVC perforation and other life-threatening risks requiring early diagnosis and in-time retrieval. Most of dislocation IVCF in the past have been shelved or removed by open surgery. It is very difficult to retrieve the filters by interventional technology.

**Case presentation:**

Here we report a 49-year-old man suffering from dislocation of IVCF implanted due to deep vein thrombosis (DVT) in the right femoral vein. Successful retrieval of the IVCF using double sheaths docking technique was done soon after confirmation of the dislocation. Importance of monitoring and early detection of dislocation of IVCF should be emphasized to avoid further complications.

**Conclusions:**

The double vascular sheaths docking technique can be considered as a preferential option in difficult operative situation.

## Background

DVT in lower extremities occurs under conditions of venous endothelial damage, blood hypercoagulation, and stasis. The main risks of DVT include early fatal pulmonary embolism (PE) and late pulmonary hypertension. For patients with anticoagulation contraindications and recurrent DVT, IVCF should be implanted to prevent fatal PE. Furthermore, monitoring on IVCF status is critical to guarantee its’ thrombus capture effect and reduce filter-derived complications.

IVCF-derived adverse events have been reported as placement issues (45.1%), IVC penetration (29.9%) and IVC filter fracture (27.1%) [1–5]. At the same time, related data suggested a high rate of serious complications after temporary IVCF implantation, calling for the urgency of strictly following indications of IVCF and carefully monitoring. Early diagnosis and in-time retrieval are important to avoid severe complications.

We report an insidious case of IVCF leg dislocation, potentially leading to perforation of the vena cava. Double sheaths docking technology was used to successfully retrieved the IVCF, verifying the importance of timely handling of IVCF migration.

## Case presentation

**Fig. 1:**
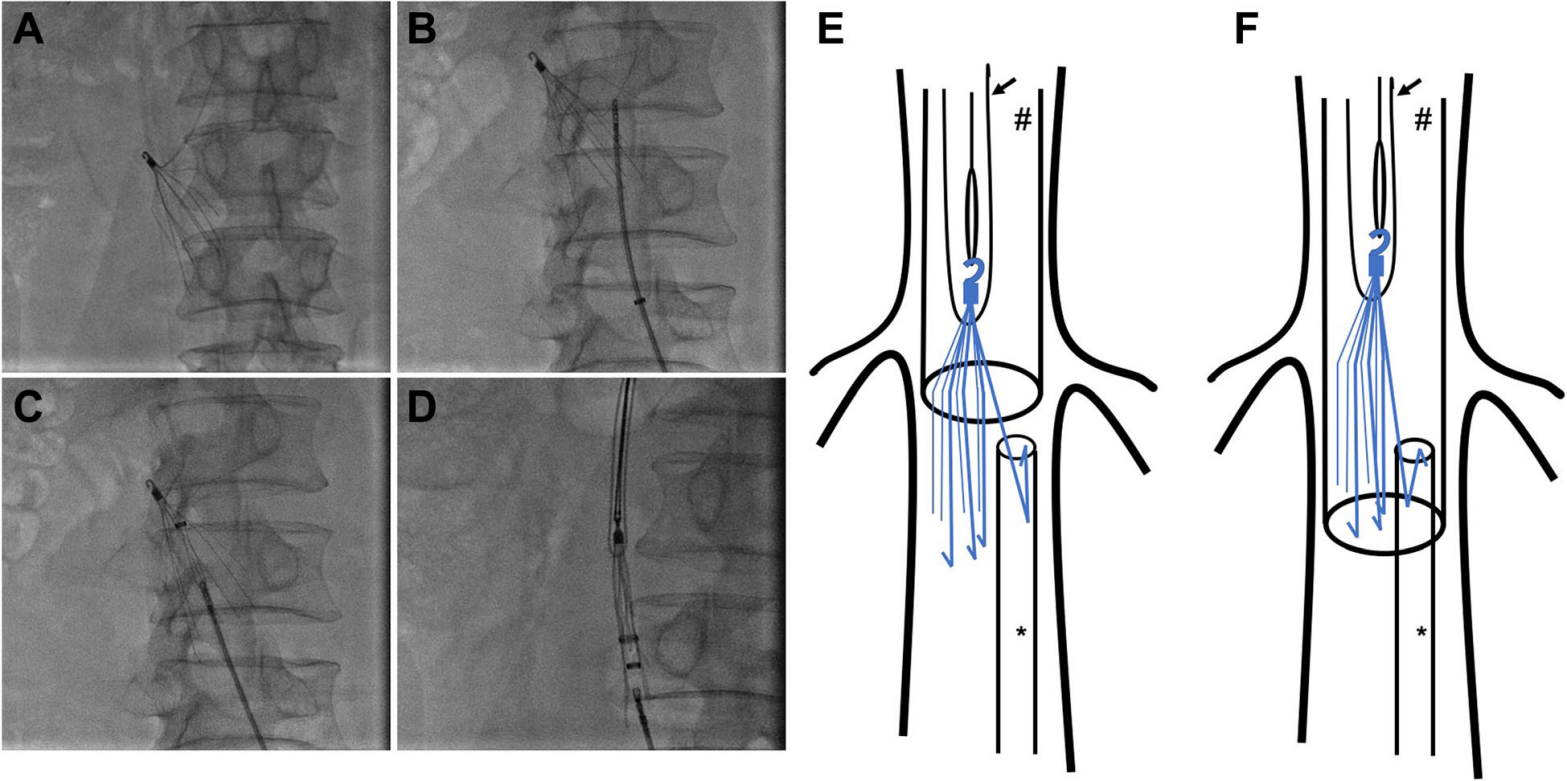
Combination of double sheaths docking technique and Loop-Snare technique to retrieve the IVC filter. (**A**) One leg of the filter is dislocated and bent cephalad. (**B**-**C**) use of disposable biopsy forceps to retrieve the dislocated filter leg into an 8F vascular sheath, (**D**) push the 13F vascular sheath downwards to complete the docking of the two vascular sheaths and retrieve the filter, (**E**-**F**) model for double sheaths docking technique to retrieve the filter. * 8F sheath; # 13F sheath; → 0.035 in. hydrophilic guide wire

A 49-year-old man with DVT in right femoral vein following ruptured cerebral aneurysm underwent retrievable IVCF (Denali, Bard, USA) implantation to prevent fatal PE. Pharmoco-mechanical thrombectomy was performed with angiojet system (Boston Scientific, MA, USA). During the surgery, a leg of the filter was found to be dislocated (Fig. 1A), which may cause perforation of the vena cava and retroperitoneal hematoma [6, 7]. With the help of disposable biopsy forceps (KLF-A, Hangzhou, China) (Fig. 1B), the dislocated filter leg was taken into an 8F vascular sheath (Fig. 1C). Then routine filter retrieval (13F) procedures were carried out via jugular vein approach but failed to trap the filter hook due to filter tilt and hook apposition to the cava wall. The “Loop-Snare Technique” [8] was used to trap the filter hook with continuous push of the 13F sheath downwards to the 8F one to complete docking of the two vascular sheaths, resulting in successful retrieval of the filter (Fig. 1D). During the retrieval procedures, the entire filter was kept in the lumen of the two vascular sheaths to avoid possible injury to the vena cava (Fig. 1E-F).

## Discussion

IVCF is an absolutely therapeutic choice for avoiding fatal PE when conventional anticoagulation is contraindicated or deemed ineffective [9]. A retrievable filter was our choice for the patient on considering the patient’s age and relatively good clinical expectation. In view of functional design, the axial stability of the temporary filter in the inferior vena cava is worse than the permanent one [2, 3], so postoperative monitoring of the filter is the key to maintain a good thrombus interception effect and avoid filter-related complications. Related studies on the long-term complications of filter concluded that most adverse events related to IVCF were device migration (35.6%), fractured parts embolization (15.9%) and IVC perforation (7.6%) [2, 3]. In our vascular center, approximately 250 patients receive IVCF implantation due to suffering DVT of lower extremities each year. The most common adverse events were the poor axial tilt and the displacement of the IVCF, resulting in the difficulty in IVCF retrieval. Hemodynamics change of the IVC due to ventilation and cardiopulmonary resuscitation has been considered as the main factors contributing to filter dislocation [10, 11]. Previous studies have reported multiple cases of cardiac tamponade caused by filter dislocation [9, 12–16] with serious consequences. Therefore, complications related to filter dislocation require prompt treatment.

This case revealed filter leg dislocation and axial migration of the filter. The dislocated filter leg may not only causes perforation of the inferior vena cava, resulting in retroperitoneal hematoma [17], and causes injure to the intestine, pancreas, aorta [18], but also leads to leg fracture of filter during retrieving of the filter. Nishikawa T reported that the dropped part of fractured filter leg was difficult to retrieve and had to be left in the vena cava [19]. However, recent studies demonstrated the safety and technically feasibility of endovascular retrieval of the filter that penetrated adjacent intestine [18] and adjacent vertebrae [20]. In our case, we successfully retrieve the dislocated IVCF combined with the filter tilting exceeds 25 degrees, which is often regarded as one of the important reasons for the difficulty of retrieval [21], the possible reasons are as follows: (1) The close follow-up allowed us to discover the displacement of the filter promptly, and the foot of the filter has not penetrated the vena cava; (2) Double vascular sheaths docking technology is beneficial to correct the axial direction of the filter. To our knowledge, this is the first case reporting the double sheaths docking to retrieve the dislocated filter, the docking with double vascular sheaths to retrieve the filter has the following advantages: (1) the dislocated filter leg was kept with the head-side orientation, avoiding damage to the vena cava by the barbed leg (2) the dislocated filter leg is protected by the vascular sheath, limiting the movement range of the leg, and avoiding the possibility of secondary dislocation of the leg. Recent study reviewed the complications of IVCF malfunction including the penetration, fracture, or migration of the device, and assessed the result of open surgery retrieval and endovascular retrieval. Comparing to the open surgery retrieval, endovascular retrieval of IVCF has a significantly lower likelihood of thromboembolic complication, and a trend to lower infectious complications, mortality, hospital costs, however, with no statistical significance [22]. Therefore, endovascular retrieval of dislocated IVCF may have obvious advantages compared with open surgery due to its minimal invasiveness and safety. This report aims to highlight the importance of postoperative filter monitoring to avoid serious filter-derived complications. The double vascular sheaths docking technique can be considered as a preferential method to retrieve dislocated IVCF.

## Conclusion

Dislocation of the IVCF is a rare and serious complication, calling for our sufficient attention. Monitoring on IVCF status is critical to guarantee its’ thrombus capture effect and reduce filter-derived complications. The double vascular sheaths docking technique can be considered as a superior method.

## Data Availability

All data analyzed during our study are included within the published article.

## Abbreviations

IVCF: Inferior vena cava filter
DVT: Deep vein thrombosis
PE: Pulmonary embolism
